# Ovarian reserve does not influence natural conception: insights from infertile women

**DOI:** 10.1007/s00404-024-07741-6

**Published:** 2024-09-28

**Authors:** Giulia Galati, Marco Reschini, Alessandra Chine’, Laura Benaglia, Paola Vigano’, Edgardo Somigliana, Paolo Vercellini, Ludovico Muzii

**Affiliations:** 1 Department of Maternal and Child Health and Urology, Sapienza University, Viale Regina Elena, 324, 00161 Rome, Italy; 2 Fondazione IRCCS Ca’ Granda Ospedale Maggiore Policlinico, ART Unit, Milan, Italy; 3 Academic Center for Research on Adenomyosis and Endometriosis, Department of Clinical Sciences and Community Health, Università degli Studi di Milano, Milan, Italy

**Keywords:** Ovarian reserve, AMH, AFC, FSH, Infertility

## Abstract

**Purpose:**

There is several albeit not univocal evidence suggesting that ovarian reserve is not related to the chance of natural pregnancy, provided that the remnant follicular pool is sufficient to ensure regular menstrual cycles. Nevertheless, available studies have some methodological limitations, and the issue cannot be considered definitively ascertained.

**Methods:**

To further address this issue, we retrospectively selected infertile women whose infertility diagnostic work-up was unremarkable (unexplained infertility-cases) and matched them by age and study period to a group of infertile women who were diagnosed with severe male infertility (controls). If ovarian reserve impacts on natural fertility, one had to expect lower ovarian reserve among women with unexplained infertility. Tested biomarkers included AMH, AFC and day 2–3 serum FSH. The primary aim was the frequency of women with serum AMH < 0.7 ng/ml.

**Results:**

Two-hundred fifty-two women with unexplained infertility and 252 women with male infertility were included. All biomarkers of ovarian reserve did not differ between the study groups. AMH levels < 0.7 ng/mL were observed in 26 (10%) women with unexplained infertility and 35 (14%) women with male infertility (*p* = 0.28). The adjusted OR was 0.76 (95% CI: 0.44–1.33). Significant differences did not also emerge when repeating this dichotomous analysis using other biomarkers and other thresholds for the definition of low-ovarian reserve.

**Conclusion:**

This study confirms that ovarian reserve is unremarkable to natural conception. Physicians and patients should be aware of this concept to avoid inappropriate counseling and undue clinical decisions.

## What does this study add to the clinical work

**Table:** 

Low ovarian reserve, as measured by AMH, does not significantly influence the chance of natural conception. Clinicians should not rely on ovarian reserve markers during fertility counselling for spontaneous conception to avoid unnecessary anxiety and interventions.	

## Introduction

There is several evidence suggesting that ovarian reserve is not related to the chance of natural pregnancy, provided that the remnant follicular pool is sufficient to ensure regular menstrual cycles. Two prospective cohort studies recruiting women before initiating pregnancy seeking failed to show any relation between serum AMH and other biomarkers of ovarian reserve and subsequent chance of natural conception [1, 2].

Hvidman et al. failed to observe any difference in serum AMH between infertile women and women of the general population [3]. Finally, we also failed to observe any relation between serum AMH and time to pregnancy in a cohort of pregnant women [4]. Nonetheless, all these studies have some methodological limitations, and the question cannot be considered definitively ascertained. Moreover, evidence is not univocal. Korsholm et al. documented higher pregnancy rate in women with regular menstrual cycle and high AMH, even the association was no more significant when adjusting for age [5].

In this study, we investigated the impact of low-ovarian reserve on natural pregnancy using a different study design. Specifically, we focused on infertile women and compared the frequency of subjects with low-ovarian reserve between those with unexplained infertility and those with a severe male cause of infertility (who are expected to reflect the general population). If low-ovarian reserve plays a role in natural conception, one should expect a higher frequency of low-ovarian reserve among women with unexplained infertility. Conversely, if no difference emerges, one should infer that the remnant ovarian reserve is unremarkable. This study design has recently been used to investigate the role of fibroids on natural fertility [6]. It has the undisputable advantage to protect findings from most confounders (the study groups are comparable in terms of reproductive history and habits).

## Materials and methods

This retrospective cohort study was conducted at the Infertility Unit of the Fondazione IRCCS Ca’ Granda Ospedale Maggiore Policlinico. Women referring to the Infertility Unit of the hospital between January 2014 and March 2020 could be included. The study was approved by the local ethical committee (Comitato Etico Milano Area 2, N. 501/2020). Given its retrospective design, selected women did not sign an informed consent form for this specific study. However, all women referring to our infertility center were invited to sign an informed consent for their data to be used for scientific research. Women who denied their consent were excluded.

Data were obtained using the locally used database Meditex (Germany). Information was then verified and collected in more details by consulting outpatients’ and inpatients’ charts. We first identified the group of women who had an unremarkable infertility work-up and whose male partners were cryptozoospermic (repeated semen analyses showing a sperm concentration below 1 million/ml). These women were considered as controls because they reflect the general population of potentially fertile women. Subsequently, we matched these subjects by age (± 1 year) and study period (6 months) in a 1:1 ratio with a group of women with unexplained infertility (cases). Ovarian reserve was not considered for selection for both study groups. Exclusion criteria were (1) previous ovarian surgery; (2) any type of ovarian cysts, with or without an indication to surgery; (3) gynecological conditions that can reduce fertility such as uterine malformations, submucosal fibroids types 0–3, endometrial polyps, endometriosis, adenomyosis, pelvic inflammatory disease or hydrosalpinx; (4) irregular menstrual cycles; (5) Incomplete infertility diagnostic workup; (6) women over 40, to exclude age-related infertility.

The infertility diagnostic work-up for infertile couples in our clinic during the study period included a gynecological examination, a transvaginal ultrasound in the proliferative phase (including AFC assessment), and serum evaluation of day 2–3 FSH, AMH and antibodies against Chlamydia trachomatis. The hystero-salpingo contrast sonography (HyCoSy) to assess tubal patency was performed in women with no other causes of infertility and a normospermic partner. Male partners perform two semen analyses that were evaluated according to World Health Organization (WHO) recommendations (WHO, 2010). All ultrasound assessments were performed by five expert physicians with at least 5 years of experience in transvaginal ultrasonography.

The primary aim of the study was the frequency of low-ovarian reserve, defined as AMH < 0.7 ng/ml. Different definitions of low-ovarian reserve were used for the secondary outcomes, i.e., AMH < 0.5 ng/ml, AMH < 1.1 ng/ml, AFC ≤ 4, AFC ≤ 7, FSH > 12 IU/mL, and FSH > 15 IU/mL. Women with unexplained infertility were considered cases (infertile subjects) while those with male infertility were considered controls (theoretical fertile women). If the null hypothesis (ovarian reserve does do not affect natural fertility) is valid, one had to expect similar frequency of women with low AMH, low AFC, and high FSH in both groups. Conversely, if ovarian reserve impacts on natural fertility, one had to expect a higher proportion among women with unexplained infertility.

We aimed at including at least 400 women (200 with severe male infertility and 200 with unexplained infertility). This sample size was calculated based on an expected proportion of women with low-ovarian reserve among controls of 10% (local data), claiming, as clinically relevant a twofold higher proportion among women with unexplained infertility, and setting type 1 and 2 errors at 0.05 and 0.20, respectively. Based on the characteristics of our local activity, we deemed that extending our retrospective investigation over a period of about 6 years (January 2014 and March 2020) could allow to reach this target. All eligible women could be selected, we did not plan to censor the number of subjects if we overcame this sample size (to allow for more robust analyses on the secondary outcomes).

Data analyses were performed using the Statistical Package for Social Sciences (SPSS, Chicago, IL, USA), version 26.0. For continuous variables, normality was assessed using Shapiro–Wilk test. Data were reported as mean ± SD, median [interquartile range] or number (percentage) and compared using Student’s *t* test, nonparametric Mann–Whitney test or Fisher’s exact test, as appropriate. For the most relevant outcomes, data were presented as Odds Ratio (OR) and 95% Confidence Interval (95% CI). Adjusted ORs (95% CI) were calculated using a regression logistic model. We included in the model age and baseline characteristics that significantly differed between the two groups.

## Results

Two hundred and fifty-two women with unexplained infertility and 252 women with male infertility were selected. Azoospermia was diagnosed in 56 cases of male infertility (22%), the remaining 196 cases had cryptozoospermia (78%). Baseline characteristics of the two groups are shown in Table 1. The median [Interquartile—IQR] day-3 serum FSH in women with unexplained and male-factor infertility was 7.2 [5.9–8.8] and 7.2 [5.9–8.5] IU/ml, respectively (*p* = 0.32). The median [IQR] serum AMH was 2.0 [1.2–3.3] and 2.2 [1.2–4.0] ng/ml, respectively (*p* = 0.50). The median [IQR] AFC was 12 [7–17] and 13 [8–18], respectively (*p* = 0.14). A statistically significant difference emerged for the duration of infertility (couples with severe male infertility were referred earlier) and BMI (slightly higher in women with male infertility). These two variables were analyzed with non-parametric statistics and reported as median [IQR]. Since the values overlapped despite the statistical significance, we decided to look more in depth into these findings and repeated the analyses using the Student *t* test. For the duration of infertility, the mean ± Standard Deviation (SD) in women with unexplained infertility and male infertility was 3.8 ± 1.9 and 3.4 ± 2.4 years, respectively (*p* = 0.02). For BMI, it was 22.2 ± 3.6 and 23.2 ± 4.3 kg/m^2^, respectively (*p* = 0.005).

**Table 1:** Baseline characteristics of the whole cohort, and separately in the two study groups

Characteristics	Whole cohort *n* = 504	Indication to IVF		
		Unexplained	Male factor	*p*
		*n* = 252	*n* = 252	
Age (years)	35 [32–37]	35 [32 37]	35 [32–37]	0.93
BMI (Kg/m^2^)	21.7 [20.0–24.4]	21.5 [19.6–23.5]	22.0 [20.2–25.7]	0.009
Duration of infertility (years)	3 [2–5]	3 [3–5]	3 [2–4]	< 0.001
FSH (IU/l)	7.2 [5.9–8.7]	7.2 [5.9–8.8]	7.2 [5.9–8.5]	0.32
AMH (IU/ml)	2.1 [1.2–3.7]	2.0 [1.2–3.3]	2.2 [1.2–4.0]	0.50
AFC	12 [8–18]	12 [7–17]	13 [8–18]	0.14
Previous surgery	67 (13%)	38 (15%)	29 (12%)	0.29
Previous pregnancies	172 (34%)	92 (37%)	80 (32%)	0.30
Previous live births	97 (19%)	45 (18%)	52 (21%)	0.50

Data are reported as median [interquartile range] or number (percentage)

AMH levels < 0.7 ng/ml were observed in 26 (10%) women with unexplained and in 35 (14%) women with male-factor infertility (*p* = 0.28). The crude and adjusted ORs for infertility in women with low AMH was 0.71 (95% CI: 0.42–1.23) and 0.76 (95% CI: 0.44–1.33), respectively. Secondary analyses with the use of different definitions of low-ovarian reserve are shown in Table 2. A statistically significant difference emerged only for AMH < 0.5 ng/ml, but this difference was in contrast with the studied hypothesis (i.e., that a detrimental impact of low-ovarian reserve would be supported by the observation of a higher frequency of low-ovarian reserve among women with unexplained infertility). Table 2 also illustrates the results of the subgroup analysis using the primary definition of low-ovarian reserve (AMH < 0.7 ng/ml) according to age (≤ and > 35 years) and duration of infertility (≤ and > 3 years). No significant results emerged.

**Table 2:** Frequency of low-ovarian reserve in the two study groups and according to different definitions

Biomarker and threshold	Indication to IVF		
	Unexplained	Male factor	*p*
	*n* = 252	*n* = 252	
AMH (ng/mL)			
AMH < 0.5	12 (5%)	25 (10%)	0.04
AMH < 0.7	26 (10%)	35 (14%)	0.28
AMH < 1.1	54 (21%)	57 (23%)	0.83
AFC			
AFC ≤ 4	16 (6%)	24 (10%)	0.25
AFC ≤ 7	64 (25%)	53 (21%)	0.29
FSH (IU/mL)^a^			
FSH > 15	3 (1%)	6 (2%)	0.34
FSH > 12	16 (7%)	10 (4%)	0.31
AMH < 0.7 ng/ml			
Age ≤ 35	12/150 (8%)	16/148 (11%)	0.43
Age > 35	14/102 (14%)	19/104 (18%)	0.45
Duration of infertility ≤ 3	12/135 (9%)	24/168 (14%)	0.16
Duration of infertility > 3	14/117 (12%)	11/84 (13%)	0.83

Data are reported as median [interquartile range] or number (percentage)

^a^Data available for 493 patients (248 unexplained and 245 male factor)

Significant differences could not also be observed when performing univariate and multivariate analyses according to pre-specified thresholds of AMH, AFC and FSH, expect for AMH < 0.5 ng/mL (Table 3). For this latter threshold, the crude and adjusted ORs for infertility in women with low AMH were 0.45 (95% CI: 0.22–0.93) and 0.44 (95% CI:0.21–0.91), respectively.

**Table 3:** Univariate and multivariate analysis according to pre-specified thresholds of AMH, AFC and FSH

	OR (95% CI)	*p*	Adjusted OR (95% CI)*	*p*
AMH (ng/mL)				
≤ 0.5	0.45 (0.22–0.93)	0.03	0.44 (0.21—0.91)	0.03
≤ 0.7	0.71 (0.42–1.23)	0.22	0.76 (0.44–1.33)	0.34
≤ 1.1	0.93 (0.61–1.42)	0.75	1.00 (0.64–1.54)	0.99
AFC				
≤ 4	0.64 (0.33–1.24)	0.19	0.68 (0.35–1.35)	0.27
≤ 7	1.28 (0.84–1.94)	0.25	1.43 (0.92–2.22)	0.11
FSH (IU/mL)				
> 15	0.49 (0.12–1.97)	0.31	0.61 (0.14–2.60)	0.50
> 12	1.62 (0.72–3.65)	0.24	1.67 (0.73–3.82)	0.23

*OR* Odds ratio for infertility, *CI* confidence interval

*OR was adjusted for woman’s age, woman BMI, and duration of infertility

## Discussion

In this study, we failed to highlight a difference in the frequency of low-ovarian reserve between women with unexplained infertility (infertile subjects) and those whose male partner had severe infertility (theoretical fertile women). This conclusion remained valid even when using different definitions of low-ovarian reserve and when focusing on subgroups based on women’s age or duration of infertility.

We only identified a paradoxical lower frequency of low-ovarian reserve among women with unexplained infertility with the use of a cut-off for AMH of 0.5 ng/ml. It remained stable in both univariate and multivariate analyses. However, this finding could be a type-I error, which refers to the likelihood of incorrectly rejecting the null hypothesis while the observed statistical significance was achieved only by chance. In fact, given the retrospective nature of this study and the multiple comparisons made, there was some inherent risk of type-I errors. To note, this observation cannot question the conclusion of our study, i.e., that low-ovarian reserve does not influence natural conception.

Our findings are in line with the current available literature on the relation between remnant ovarian reserve and natural conception. The confirmation of the worthlessness of ovarian reserve with the use of a different and original study design further toughens up the conclusion. Up to now, two pivotal cohort studies highlighted the irrelevance of low-ovarian reserve for natural conception [1, 2]. In both studies, biomarkers of ovarian reserve were assessed prior to initiate pregnancy seeking and low-ovarian reserve was related to the chance of natural conception. The sample size was huge (1202 and 981 women, respectively). In the first study, the adjusted Hazard Ratio (HR) of natural conception in women with low-ovarian reserve (AMH < 1.0 ng/ml) was 1.13 (95% CI: 0.85–1.49) [1]. In the second study, the adjusted OR for women with low AMH (< 0.7 ng/ml) was 1.19 (95% CI: 0.88–1.61) [2]. This study design is outstanding but has some limitations. They include the recruitment of women prone to enter clinical trials (who may not reflect the general population), the necessity to adjust for age (introducing therefore an arbitrary statistical modeling), the outcome of clinical pregnancy rather than live birth and the lack of information on the male partner. For this reason, the fundamental notion that low-ovarian reserve does not influence natural conception needed evidence from other study designs. Another different methodological approach to investigate this issue was proposed by Somigliana et al. [4]. In that study, the authors recruited pregnant women at the time of the first-trimester screening for aneuploidy and selected those who needed more than 12 months to conceive (subfertile women, *n* = 76) and matched them by age to a control group of women conceiving in less than 1 year. The frequency of low AMH (< 1.1 ng/ml) was similar in the two groups (15% and 20%, respectively). The adjusted OR for subfertility in women with low AMH was 0.85 (95% CI 0.35–2.10) [4]. These findings overlap with another small study that related serum AMH to time to pregnancy in a group of 84 women [7]. In addition, it is worth discussing the study design from Hvidmann et al. [3] because it has some overlaps with our design. These authors recruited 382 infertile women and compared biomarkers of ovarian reserve with those obtained in an independent control group of 350 non-users of hormonal contraception with no history of infertility and similar age who were recruited in a prospective cross-sectional study among female health care workers. They also failed to show any difference in serum AMH, but the study has some flaws [3]. Of utmost relevance is the comparison of two very different populations. In this regard, our study design is more reliable because both cases and controls were infertile and selection biases are unlikely. Finally, in contrast to previous evidence, Korsholm et al. documented higher pregnancy rate in women with regular menstrual cycle and high AMH. The authors evaluated natural conception in a cohort of 260 women who initiated an attempt to become pregnant naturally or achieve unplanned natural pregnancy. They related the event of pregnancy to AMH assessed before entering the study. However, the significant association got lost when adjusting for age [5].

The notion that remnant ovarian reserve is unremarkable to natural conception is jarring and counter-intuitive for general gynecologists. However, it is fundamental to endorse this notion to avoid undue counseling as well as overdiagnosis and over-treatment to the general population and to infertile women [8, 9]. To illustrate how spread is the erroneous notion that ovarian reserve can influence natural conception, the Italian Ministry of health includes low-ovarian reserve as a cause of infertility among indications to Assisted Reproductive Technologies (ART). This is paradoxical considering that low-ovarian reserve is conversely an obstacle to ART success [10]. The impact on the general population is potentially devastating. The current spread of AMH testing may generate anxiety and undue stress, as well as over-treatment. Counseling practices for patients need to be revised to reduce the spread of this frightening belief. Low AMH levels should no longer be considered as an indicator of reduced chances for natural conception. On the contrary, patients with low AMH levels may be reassured by explaining that AMH should not be used to evaluate fertility. Being diagnosed with low-ovarian reserve is inevitably troublesome, but affected women must be aware that this condition could be detrimental only if they will not conceive naturally and will need ART or if they will not attempt to become pregnant in the short-medium term. This second point is intuitive (ovarian reserve unreluctantly reduce with age) but, again, not strongly supported. Of relevance here is that low AMH is not even a reliable predictor of early menopause [11, 12]. In a recent population-based survey performed in Australia, it emerged that almost half of the women performed AMH for unsupported reasons: understanding their chance of getting pregnant (19%), finding out if a medical condition has affected their fertility (11%), curiosity about their fertility (9%), and considering delaying pregnancy (2%) [8]. A recent international investigation on the information provided by websites that sell direct-to-consumer AMH testing showed that most of them included false and misleading claims on the capacity of AMH to predict fertility potential and age at menopause [9]. On the contrary, AMH should not be routinely measured to get information about the woman’s reproductive potential. Furthermore, women with only low AMH levels and a partner with normal semen analysis, who have been trying to conceive for less than 6–12 months, should not be prematurely scheduled to ART. The incremental benefit of ART is reduced if the ovarian reserve is low and the rate of worsening of ovarian reserve is not predictable. Overall, prescription of biomarkers of ovarian reserve should be considered only in women with an indication to ART to counsel them regarding the chances of success of the procedure and to tailor the regimen of ovarian stimulation.

Our study design may surprise at prima face. We indeed recruited both cases and controls from a population of infertile women. On the other hand, it has some fundamental advantages. First, the two groups have similar characteristics and, therefore, our results are less exposed to selection biases. Second, we applied strict selection criteria to reduce confounders. The exclusion of women older than 40 should has eliminated the rate of women with age-related infertility. We also excluded women with any known causes of infertility in both groups, i.e., those with a history of ovarian surgery, those with irregular menstrual cycles and those who were diagnosed with ovarian cysts, uterine malformations, endometrial polyps, endometriosis, adenomyosis, pelvic inflammatory disease or hydrosalpinx, and submucosal fibroid types 0–3. This choice was taken to limit confounders. We were exclusively interested in women with pure severe male infertility and in those with unexplained infertility. Moreover, all participants were examined at the same center using the same algorithm and ultrasound equipment, laboratory and AMH assay.

On the other hand, some limitations must be recognized. First, the retrospective design inevitably exposes our findings to potential inaccuracies and biases, such as recall bias, selection bias, and information bias. In retrospective studies, the accuracy of the collected data depends heavily on the completeness and reliability of existing records. However, the crucial data used for this study (serum AMH, AFC and serum FSH) were obtained within the context of a systematic and standardized clinical and ultrasound assessment. For this reason, the intrinsic limitation of the retrospective design should be tempered.

To mitigate the impact of these limitations, several steps were taken. First, we ensured that all data were extracted from a single, well-maintained database, minimizing the risk of incomplete or inconsistent records. Second, the clinical and ultrasound assessments were conducted according to standardized protocols, reducing variability and enhancing the reliability of the recorded data. Third, we performed a thorough validation of the data to identify and correct any discrepancies or errors.

Despite these measures, it is essential to acknowledge that some degree of bias and inaccuracy is unavoidable in retrospective studies. Therefore, our findings should be interpreted with caution, and future prospective studies are warranted to confirm our results and to provide a more comprehensive understanding of the subject matter.

The second limitation of this study is that misdiagnoses may have occurred. We may have erroneously included, among women with unexplained infertility, some cases with undetected causes of infertility such as mild/minimal endometriosis. Even if the performance of an in-depth sonography allowed us to exclude advanced forms of the disease, some cases of early endometriosis with only superficial peritoneal lesions could have been missed [13]. In this context, one may consider the possibility to have erroneously included infertile women also among the controls (the male-factor cases). Of note, women with an infertile partner did not undergo hystero-salpingo contrast sonography to assess tubal patency. Overall, however, even if misdiagnoses may have occurred, this event is expected to affect less than 10% of women [14]. Third, even if we overcome the scheduled sample size, the number of selected cases is relatively small, and this may affect the precision of the results of subgroup analyses. Larger multicenter studies using a similar study design are needed to confirm our results.

In conclusion, our findings confirm the notion that low-ovarian reserve does not influence natural conception. We plea for a wilder diffusion of this notion among physicians and patients to avoid undue anxiety and distress, as well as overdiagnosis and overtreatment. To date, ovarian reserve assessment is justified only in women scheduled for ART.
